# Tamoxifen in the Mouse Brain: Implications for Fate-Mapping Studies Using the Tamoxifen-Inducible Cre-loxP System

**DOI:** 10.3389/fncel.2016.00243

**Published:** 2016-10-20

**Authors:** Martin Valny, Pavel Honsa, Denisa Kirdajova, Zdenek Kamenik, Miroslava Anderova

**Affiliations:** ^1^Department of Cellular Neurophysiology, Institute of Experimental Medicine, Academy of Sciences of the Czech RepublicPrague, Czech Republic; ^2^2nd Faculty of Medicine, Charles UniversityPrague, Czech Republic; ^3^Laboratory for Biology of Secondary Metabolism, Institute of Microbiology, Academy of Sciences of the Czech RepublicPrague, Czech Republic

**Keywords:** tamoxifen, brain metabolism, fate-mapping, Cre-loxP, gene-targeting

## Abstract

The tamoxifen-inducible Cre-loxP system is widely used to overcome gene targeting pre-adult lethality, to modify a specific cell population at desired time-points, and to visualize and trace cells in fate-mapping studies. In this study we focused on tamoxifen degradation kinetics, because for all genetic fate-mapping studies, the period during which tamoxifen or its metabolites remain active in the CNS, is essential. Additionally, we aimed to define the tamoxifen administration scheme, enabling the maximal recombination rate together with minimal animal mortality. The time window between tamoxifen injection and the beginning of experiments should be large enough to allow complete degradation of tamoxifen and its metabolites. Otherwise, these substances could promote an undesired recombination, leading to data misinterpretation. We defined the optimal time window, allowing the complete degradation of tamoxifen and its metabolites, such as 4-hydroxytamoxifen, *N*-desmethyltamoxifen, endoxifen and norendoxifen, in the mouse brain after intraperitoneal tamoxifen injection. We determined the biological activity of these substances *in vitro*, as well as a minimal effective concentration of the most potent metabolite 4-hydroxytamoxifen causing recombination *in vivo.* For this purpose, we analyzed the recombination rate in double transgenic Cspg4-cre/Esr1/ROSA26Sortm14(CAG-tdTomato) mice, in which tamoxifen administration triggers the expression of red fluorescent protein in NG2-expressing cells, and employed a liquid chromatography, coupled with mass spectrometry, to determine the concentration of studied substances in the brain. We determined the degradation kinetics of these substances, and revealed that this process is influenced by mouse strains, age of animals, and dosage. Our results revealed that tamoxifen and its metabolites were completely degraded within 8 days in young adult C57BL/6J mice, while the age-matched FVB/NJ male mice displayed more effective degradation. Moreover, aged C57BL/6J mice were unable to metabolize all substances within 8 days. The lowering of initial tamoxifen dose leads to a significantly faster degradation of all studied substances. A disruption of the blood-brain barrier caused no concentration changes of any tamoxifen metabolites in the ipsilateral hemisphere. Taken together, we showed that tamoxifen metabolism in mouse brains is age-, strain- and dose-dependent, and these factors should be taken into account in the experimental design.

## Introduction

Gene targeting has proven to be a powerful tool for precise manipulation of the mammalian genome, by generating thousands of mutant mouse strains. Studies of these mutant mice have yielded extremely useful information in all fields of biological sciences, and theoretically, gene targeting can be used to generate mutant mice for all genes in the near future. However, many genes are essential for development and their mutations result in pre-adult lethality, preventing further studies of their functions in adulthood (Weinstein et al., 2000; Deng, 2002; Coumoul and Deng, 2003; Friedberg and Meira, 2006). In the past decades, Cre-loxP technology, combined with tamoxifen-inducible systems, has been used to overcome this pre-adult lethality (Le and Sauer, 2000). In addition, this technology has enabled precise gene manipulation in distinct cell subpopulations at any specific time point, which is known as temporally and spatially controllable gene expression. Besides gene knockout, Cre-loxP technology has enabled labeling of any cell types, and following their fate, which is called genetic fate-mapping technique. The main inducer of these genetic modifications is tamoxifen, which enables enzymatic activity of Cre-mutated estrogen receptor (Cre-ER^TM^) fusion protein, and therefore knowledge of tamoxifen administration, metabolism and degradation in a living organism is of great importance. There exist several ways of tamoxifen administration (orally, intraperitoneally, and in drinking water) with different advantages and disadvantages, and simultaneously, fundamental ways of metabolism and degradation of tamoxifen and its metabolites are already known. Despite the fact that a great part of all genes are expressed in the central nervous system (CNS) and numerous genetic modifications target various cells in the nervous tissue, only a limited amount of information regarding tamoxifen metabolism in this organ is available. Since many processes taking place in the CNS show different properties when compared to the rest of the organism (e.g., due to the presence of the blood-brain barrier, high vascularization, immune privileged tissue, different enzymes etc.) tamoxifen metabolism could also be different, and therefore, the tamoxifen-dependent recombination rate could be altered when compared to *in vitro* conditions or other organs.

In the present study, we analyzed the degradation kinetics of tamoxifen and its metabolites in mouse brains after intraperitoneal administration, and we focused on the variances among mice of different age, sex or strain background. Simultaneously, we also examined different application schemes and doses of tamoxifen. The aim of this study is to define the tamoxifen administration scheme, which will enable the maximal recombination level together with minimal animal mortality. Additionally, we also focused on tamoxifen degradation kinetics, because for all genetic fate-mapping studies, the period during which tamoxifen or its metabolites remain active in the CNS, is essential.

## Materials and Methods

### Animals

All procedures involving the use of laboratory animals were performed in accordance with the European Communities Council Directive, 24 November 1986 (86/609/EEC) and animal care guidelines approved by the Institute of Experimental Medicine, Academy of Sciences of the Czech Republic (Animal Care Committee on April 7, 2011; approval number 018/2011). All efforts were made to minimize both the suffering and the number of animals used. Experiments were performed on young adult (60–90 days) or aged (over 15 months) C57BL/6J and FVB/NJ mice. For the purpose of cell quantification we used transgenic mice, which were derived by crossing the mouse strain B6.Cg-Tg(Cspg4-cre-Esr1^∗^)BAkik/J and B6;129S6-Gt(ROSA)26Sortm14 (CAG-tdTomato)Hze/J (further termed Cspg4/Tomato mouse), (Jackson Laboratory, Bar Harbor, ME, USA), in which the expression of tamoxifen-inducible Cre recombinase is controlled by the Cspg4 promoter (Zhu et al., 2011). After tamoxifen/4-hydroxytamoxifen administration, tomato red fluorescent protein is expressed in Cspg4-positive cells – predominantly in NG2 glia and cells derived therefrom, e.g., differentiating oligodendrocytes and moreover in a small subpopulation of pericytes (Supplemental Figure S1A).

### Tamoxifen and Its Derivatives Solutions

For all *in vitro* experiments the tamoxifen and its derivatives were dissolved in ethanol (96%, Sigma–Aldrich, St. Louis, MO, USA). For *in vivo* experiments we used tamoxifen (20 mg/ml, Toronto Research Chemicals INC, Toronto, ON, Canada) dissolved in corn oil (Sigma–Aldrich, St. Louis, MO, USA) or 4-hydroxytamoxifen (20 mg/ml, Toronto Research Chemicals INC, Toronto, ON, Canada) dissolved in ethanol (96%, Sigma–Aldrich, St. Louis, MO, USA) and further diluted in corn oil to desired concentration.

### Induction of Focal Cerebral Ischemia in Young Adult Mice

Prior to the induction of focal cerebral ischemia (FCI), the young adult mice were anesthetized with 3% isoflurane (Abbot, Abbott Park, IL, USA) and maintained in 2% isoflurane using a vaporizer (Tec-3, Cyprane Ltd, Keighley, UK). A skin incision between the orbit and the external auditory meatus was made, and a 1–2 mm hole was drilled through the frontal bone, 1 mm rostrally to the fusion of the zygoma and the squamosal bone, and about 3.5 mm ventrally to the dorsal surface of the brain. The middle cerebral artery (MCA) was exposed after the dura was opened and removed. The MCA was occluded by short coagulation with bipolar tweezers (SMT, Czech Republic), at a proximal location, followed by transection of the vessel to ensure permanent occlusion. During the surgery, body temperature was maintained at 37 ± 1°C using a heating pad. This MCA occlusion (MCAo) model yields small infarct lesions in the parietal cortical region (Honsa et al., 2013).

### Preparation of Tomato-Positive Cell Cultures from the Neonatal Mouse Brain

Neonatal 0–3 days old Cspg4/Tomato mice were decapitated, whole brains were dissected out and mechanically dissociated by gentle trituration using 1 ml pipette in DMEM/F12 medium, with penicillin/streptomycin (all from Invitrogen, Carlsbad, CA, USA), 20 ng/ml PDGFα (Peprotech, Rocky Hill, NJ, USA), 4 mM glutamine (Sigma–Aldrich, St. Louis, MO, USA) and 15% fetal bovine serum (HyClone, Thermo Scientific, Waltham, MA, USA), further referred to as basal medium. The resulting cell suspension was cultured on the poly-L-lysine-coated cover slips (PLL, Sigma–Aldrich, St. Louis, MO, USA), in a humidified atmosphere with 5% CO_2_ at 37°C, and maintained in basal medium supplemented with tamoxifen/4-hydroxytamoxifen/*N*-desmethyltamoxifen/(E/Z)-*N*-desmethyl-4-hydroxytamoxifen (endoxifen)/(E/Z)-*N,N*-didesmethyl-4-hydroxytamoxifen(norendoxifen) (Toronto Research Chemicals INC, Canada), in concentrations ranging from 0.5 ng/ml to 2000 ng/ml. After 3 days of cultivation, the cover slips were used for quantification of tomato-positive (tomato+) cells.

### Cell Quantification

For quantification of tomato+ cells, mice were treated with 4-hydroxytamoxifen in two doses [0.05 to 40 mg/kg intraperitoneally (i.p.)] for two consecutive days. Two days after the second 4-hydroxytamoxifen injection, mice were deeply anesthetized with pentobarbital (100 mg/kg, i.p.) and perfused transcardially with 20 ml of saline followed by 20 ml of cooled 4% paraformaldehyde (PFA), in 0.1 M phosphate buffer (PB). Brains were dissected out, post-fixed overnight with PFA, and treated with a sucrose gradient (ranging from 10 to 30%) for cryoprotection. Coronal 30 μm-thick slices were prepared using a cryostat (Leica CM1850, Leica Microsystems, Wetzlar, Germany). Cover slips with cell cultures were fixed for 10 min using 4% PFA in 0.1 M PB. All chemicals were purchased from Sigma–Aldrich (St. Louis, MO, USA). The brain slices, along with fixed cover slips were mounted using Aqua Poly/Mount (Polysciences Inc., Eppelheim, Germany). A Zeiss 510DUO LSM confocal microscope equipped with Ar/HeNe lasers and 20x objective was used for histo-/cyto-chemical analyses. For quantification of cultivated cells, at least three independent cultivations for each concentration of tamoxifen and its metabolites were used. For quantification of tomato+ cells in brain slices, 1–3 mice were used for particular concentration of administrated 4-hydroxytamoxifen.

### Liquid Chromatography-Mass Spectrometry

#### Sample Preparation

For determination of brain/blood concentration of tamoxifen and its metabolites, tamoxifen was administered to mice in one/two/five doses (100/200 mg/kg i.p.) 24 h apart. After 6 h, 2, 4, 6, and 8 days the mice were decapitated, and the brains were quickly dissected out/blood samples were collected and frozen at -80°C until sample processing. In the case of mice with FCI, tamoxifen was administrated in two doses (200 mg/kg i.p.) 24 h apart, and 3 days after the second tamoxifen injection, the FCI was induced, and 1 day after FCI induction, the brains were isolated. According to (Lien et al., 1991), the brains were homogenized (1:5, w:v) in ice cold 50 mM Tris-HCl (Sigma–Aldrich, St. Louis, MO, USA), pH 7.4 using a tissue ruptor homogeniser (Qiagen, Hilden, Germany). Brain homogenates were mixed with acetonitrile (1:1, v:v) and precipitated proteins removed by centrifugation (15 000 *g* for 6 min). The supernatants represented extracts for analysis by liquid chromatography coupled with mass spectrometry (LC-MS). The same procedure applied to tamoxifen non-treated mice was used to prepare analyte-free matrix. For each analyzed time-point we used 3–5 mice with the exception of aged mice treated with full dose of tamoxifen at time-point 2 days. At this time-point we analyzed two mice and due to high mortality of aged mouse we reduced the initial tamoxifen dose in the next experiments with aged mice.

#### Chemicals and Standard Solutions

Standards of tamoxifen, 4-hydroxytamoxifen, *N*-desmethyltamoxifen, (E/Z)-*N*-desmethyl-4-hydroxytamoxifen(endoxifen), (E/Z)-*N,N*-didesmethyl-4-hydroxytamoxifen (norendoxifen), and (Z)-4-hydroxytamoxifen-d5 were purchased from Toronto Research Chemicals INC (Toronto, ON, Canada). The ratio of Z and E isomers in *N*-desmethyl-4-hydroxytamoxifen (48% of Z isomer) and *N,N*-didesmethyl-4-hydroxytamoxifen (66% of Z isomer) was determined by liquid chromatography under isocratic conditions (35% B) with UV detection at 245 nm (further conditions are described below in the LC-MS analysis paragraph). The standards were dissolved in acetonitrile, at the concentration of 200 μg/ml and diluted with analyte-free matrix at the required concentration.

#### Calibration Curves and Validation

A validated method (Teunissen et al., 2011) for determination of tamoxifen and its main phase I metabolites was adopted for analysis of the samples. Four five-point calibration curves were constructed: (1) From limit of detection (LOD) (0.625 or 1.250 ng/ml) to 10 ng/ml; (2) from 5 to 160 ng/ml; (3) from 62.5 ng/ml to 1000 ng/ml; and (4) from 1 to 64 μg/ml. Calibration solutions, quality control (QC) samples and extracts for calibrations (3) and (4) were, prior to analysis diluted 100x with the analyte-free matrix. The calibration curves were fitted linearly or quadratically with the residual values lower than 15% (20% for a limit of quantification – LOQ – level). QC samples were measured in triplicates at the LOQ, low, middle and high concentration levels of the respective calibration curve. Recovery values of the QC samples between 85 and 115% (80–120% for LOQ) and relative standard deviation lower than 5% for all QC samples and all analytes demonstrated satisfactory accuracy and precision of the method. LOQ values were determined as follows: 1.25 ng/ml for 4-hydroxytamoxifen and *N*-desmethyl-4-hydroxytamoxifen; 0.625 ng/ml for tamoxifen, *N*-desmethyl-tamoxifen, and *N,N*-didesmethyl-4-hydroxytamoxifen. LOD values were determined as 0.313 ng/ml for all analytes.

#### LC-MS Analysis

(Z)-4-Hydroxytamoxifen-d5 was used as the internal standard for all determined analytes, at the final concentration of 2.5 (calibrations 1 and 3) or 40 ng/ml (calibrations 2 and 4). The extracts, calibration or QC samples were centrifuged (13 000 rpm, 5 min) and mixed with the solution of internal standard in acetonitrile [9:1 (v:v)] or diluted 100x with analyte-free extract and then mixed with the solution of internal standard in acetonitrile [9:1, (v:v)]. The samples were analyzed on an Acquity UPLC system with an LCT premier XE time-of-flight mass spectrometer (Waters, Milford, MA, USA) using the liquid chromatography column Acquity UPLC BEH C18 (50 mm × 2.1 mm I.D., particle size 1.7 μm, Waters, Milford, MA, USA) and a two-component mobile phase. The mobile phase A and B consisted of 0.1% formic acid in water and acetonitrile, respectively. The analyses were performed under a linear gradient program (min/%B) 0/30, 2/30, 6.5/52.5 followed by a 1 min column clean-up (95% B) and a 1.5 min equilibration (30% B). The total analysis time was 9 min. The column temperature was set at 30°C, and flow rate at 0.4 ml/min. The mass spectrometer operated in the V mode, the capillary voltage was set at +2600 V; cone voltage, +40 V; desolvation gas temperature, 350°C; ion source block temperature, 120°C; cone gas flow, 50 L/h; desolvation gas flow, 800 L/h; ion guide 1 and 2 RFs, 200 and 300 V, respectively; hexapole RF, 150 V. Ions in the m/z range 100–1000 were detected using a scan time of 0.15 s and an inter-scan delay of 0.01 s. The mass accuracy was kept below 5 ppm using lock spray technology, with leucine enkephalin as the reference compound (2 ng/μl, 5 μl/min). The data were processed with MassLynx software using the QuanLynx application manager (Waters, Milford, MA, USA). The chromatograms of ions corresponding to [M+H]^+^ adducts were extracted with the tolerance window of 0.05 Da, and smoothed (mean method, two smoothing iterations, width of 2 scans).

### Statistics

The results are expressed as the mean ± SEM. Statistical analyses of the differences among groups were performed using one-way ANOVA for multiple comparisons with Tukey’s *post hoc* test and Student’s *t*-test when appropriate. Values without the error bars were under the LOD. Values of *p* < 0.05 were considered significant, *p* < 0.01 very significant and *p* < 0.001 extremely significant.

## Results

In this study, we aimed to clarify the degradation kinetics of tamoxifen and its metabolites in the mouse brain in the context of genetic fate-mapping using Cre-loxP systems. Since, to the best of our knowledge, no work showing how long tamoxifen and its active metabolites remain in the brain in effective concentration is available, we wanted to define the time window allowing complete degradation of tamoxifen and its metabolites. This is of particular importance regarding the experiments in which the cell fate is influenced by any treatment, such as surgery or drug administration. The time window between tamoxifen administration and the treatment should be large enough to allow complete degradation of tamoxifen and its metabolites, otherwise they might promote recombination in the cells that start to express the Cre recombinase, as a result of the treatment, which can lead to data misinterpretation. We first tested the efficacy of tamoxifen and its metabolites in our Cre-loxP system – Cspg4/tomato mice – *in vitro*, and subsequently we determined the effective brain concentration of the most potent metabolite 4-hydroxytamoxifen, and degradation kinetics of tamoxifen and its metabolites *in vivo*.

### Biological Activity of Tamoxifen and Its Metabolites *In vitro*

**FIGURE 1:**
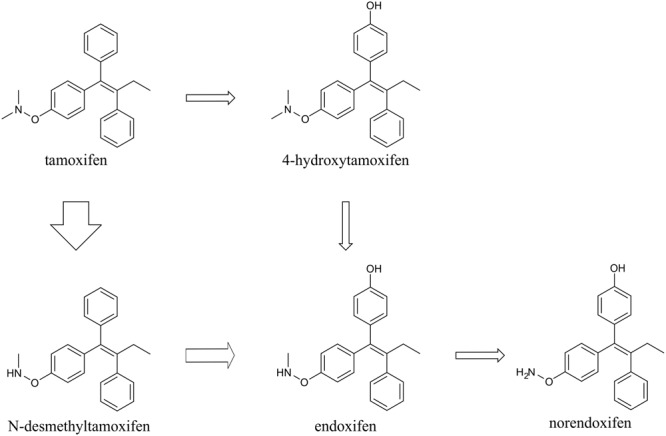
**Scheme of tamoxifen metabolism.** Tamoxifen is metabolized mainly via *N*-demethylation into *N*-desmethyltamoxifen, and subsequently endoxifen and norendoxifen. Minority of tamoxifen is 4-hydroxylated to form 4-hydroxytamoxifen, which can be further transformed to endoxifen. The thickness of the arrows indicates the major direction of tamoxifen conversion.

Tamoxifen, is extensively metabolized *in vivo*, in hepatic cells, predominantly by cytochrome P450 system via two pathways, 4-hydroxylation and *N*-demethylation. The majority of tamoxifen is transformed to *N*-desmethyltamoxifen, and subsequently to endoxifen and norendoxifen. The rest is metabolized to 4-hydroxytamoxifen, which can also be transformed to endoxifen in the next step (Jordan, 2007) (**Figure 1**).

**FIGURE 2:**
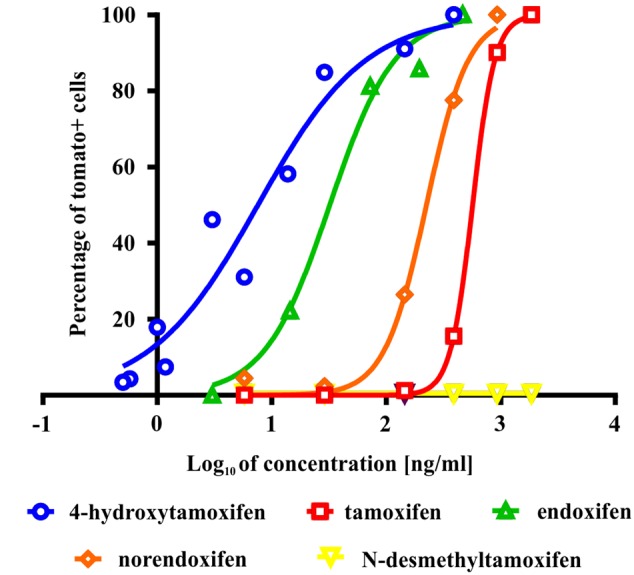
**Effectivity of tamoxifen and its metabolites in promoting recombination *in vitro*.** The most potent metabolite of tamoxifen is 4-hydroxytamoxifen with EC = 7.6 ± 2.5 ng/ml, followed by endoxifen (EC = 32.1 ± 1.6 ng/ml), norendoxifen (EC = 224.5 ± 20.4 ng/ml), tamoxifen itself (EC = 553.4 ± 68.4 ng/ml) and inactive *N*-desmethyltamoxifen.

To test the ability of tamoxifen and its metabolites to promote recombination in Cspg4/tomato mice, we isolated cells from the brains of neonatal Cspg4/tomato mice, cultivated them with different concentrations of tamoxifen or its metabolites, and subsequently, quantified the number of recombined (i.e., tomato+) cells. The highest capability to promote recombination, possess 4-hydroxytamoxifen with EC_50_ of 7.6 ± 2.5 ng/ml (Supplemental Figure S1B), followed by endoxifen (EC_50_ = 32.1 ± 1.6 ng/ml), norendoxifen (EC_50_ = 224.5 ± 20.4 ng/ml), tamoxifen (EC_50_ = 553.4 ± 68.4 ng/ml) and *N*-desmethyltamoxifen, which, despite being the main metabolite of tamoxifen, showed no biological activity within the range of concentrations tested (**Figure 2**). These data correspond well with previous studies and confirm the outstanding efficacy of 4-hydroxytamoxifen when compared to other tamoxifen metabolites (**Table 1**) (Katzenellenbogen et al., 1984; Desta et al., 2004; Jordan, 2007).

**Table 1:** Comparison of biological activity of tamoxifen and its metabolites determined in this study and in Katzenellenbogen et al. (1984), Desta et al. (2004), and Jordan (2007).

	This study	Desta et al., 2004; Jordan, 2007	Katzenellenbogen et al., 1984
	Cells isolated from Cspg4/Tom mouse	Rat uterus/MCF-7 breast cancer cell line	MCF-7 breast cancer cell line
	
	Affinity for Cre-ERTM (ability to promote recombination) [%]	Affinity for estrogen receptor/suppression of cell growth [%]	Affinity for estrogen receptor/suppression of cell growth [%]

Tamoxifen	100	100	100
4-Hydroxytamoxifen	∼7000	∼3000–10000	∼12400
Endoxifen	∼1700	∼3000–10000	—
Norendoxifen	∼250	—	—
*N*-desmethyltamoxifen	Ineffective	—	—

### Degradation Kinetics of Tamoxifen and Its Metabolites *In vivo*

Since the metabolism rate may vary depending on different gender, strain, age and initial tamoxifen dose, we followed the fate of administrated tamoxifen in mouse brains in three sets of experiments. In the first set, we assessed the gender-, strain-, and age-related differences. In the second set of experiments we evaluated the changes caused by different dosing, and in the third set we tested the influence of FCI on the concentration of tamoxifen and its metabolites in the brain.

**FIGURE 3:**
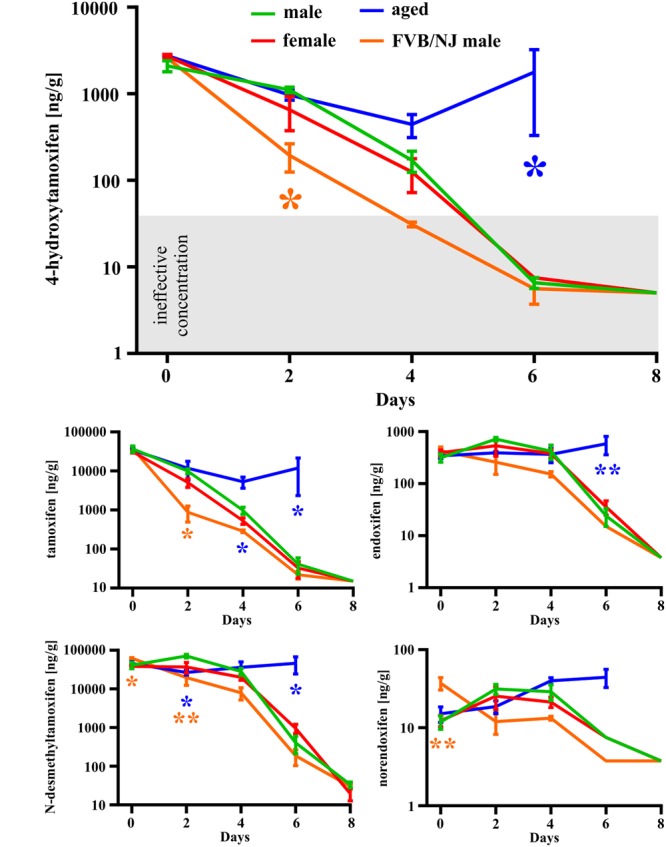
**Gender-, strain- and age-dependent differences in degradation of tamoxifen and its metabolites.** Degradation of tamoxifen and its metabolites is significantly affected by age and strain of the mice. Significances are expressed above the *x*-axis by an asterisk in the color of the respective group relative to the male group (green line). Values of *p* < 0.05 were considered significant (^∗^), p < 0.01 very significant (^∗∗^).

In the first set of experiments, we used a total dose of 400 mg/kg of tamoxifen, divided into two doses of 200 mg/kg, applied 24 h apart. Such a dose was selected as an approximate average of doses used in many studies using Cre-loxP systems (Leone et al., 2003; Kang et al., 2010, 2013; Li et al., 2010; De Biase et al., 2011; Komitova et al., 2011; Clarke et al., 2012; Benner et al., 2013; Robins et al., 2013). We chose the 2-doses scheme since in our hands use of just one dose of 200 mg/kg of tamoxifen resulted in lower recombination rate when compared to two doses (Supplemental Figure S1C). The brain concentration of tamoxifen and its metabolites was analyzed 6 h and 2, 4, 6, and 8 days after the second tamoxifen application. As depicted in **Figure 3** the degradation kinetics of tamoxifen and 4-hydroxytamoxifen were exponential-like, whereas those of endoxifen, *N*-desmethyltamoxifen and norendoxifen were characterized by a plateau phase preceding the exponential-like phase. This plateau phase is probably caused by faster production rate of endoxifen, *N*-desmethyltamoxifen and norendoxifen compared to their degradation rate as is shown in **Figure 1**. All substances were completely metabolized within 8 days with an exception of *N*-desmethyltamoxifen. Noteworthy, the peak brain concentration of tamoxifen, 6 h after the second application was 36792.0 ± 6891.8 ng/g, which represents 9.2 ± 1.7% of the total tamoxifen dose applied, and more importantly, the peak brain concentration of 4-hydroxytamoxifen was 2098.0 ± 304.5 ng/g, which is only 0.5% of the active metabolite from the total tamoxifen dose. Although no differences were observed in degradation kinetics between young adult male and female mice of the same strain, we detected differences in degradation kinetics between mice of different strains. The degradation kinetics of FVB/NJ mice is slightly, but significantly, faster than that of the C57BL/6J mice, which is further documented by a steeper plateau phase in the degradation kinetics of endoxifen, *N*-desmethyltamoxifen and norendoxifen in the case of FVB/NJ mice (**Figure 3**). The last, but most striking finding from this set of experiments is the fact that aged (over 15 months) C57BL/6J mice were almost unable to degrade all substances within 6 days. In fact, the majority of old mice died before we could analyze them, which is the reason for the missing 8 days time-point in our analysis.

**FIGURE 4:**
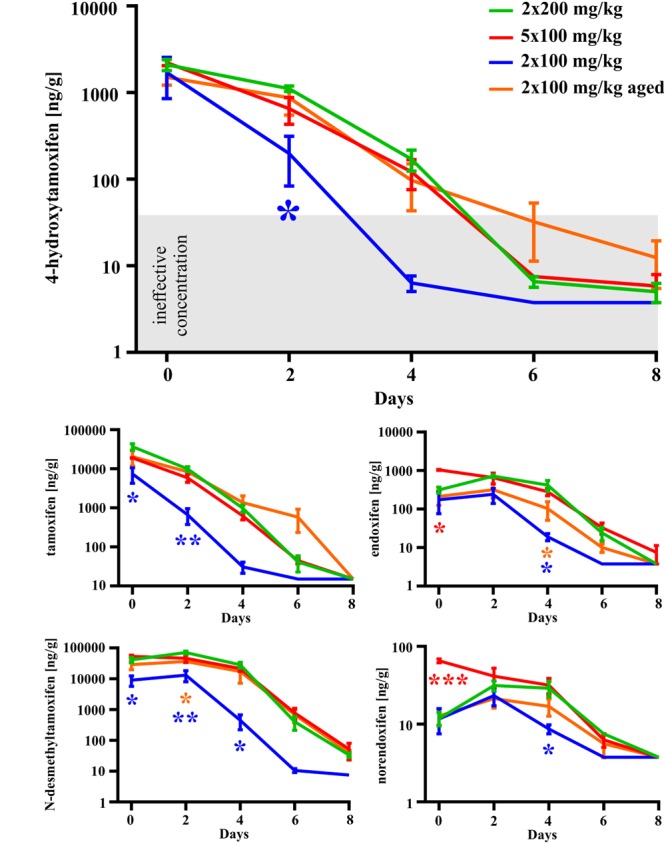
**Dose-dependent differences in degradation of tamoxifen and its metabolites in the mouse brains.** Degradation of tamoxifen and its metabolites is significantly affected by initial tamoxifen dose. Significances are expressed above the *x*-axis by an asterisk in the color of the respective group relative to 2 × 200 mg/kg group (green line). Values of *p* < 0.05 were considered significant (^∗^), *p* < 0.01 very significant (^∗∗^) and *p* < 0.001 extremely significant (^∗∗∗^).

**FIGURE 5:**
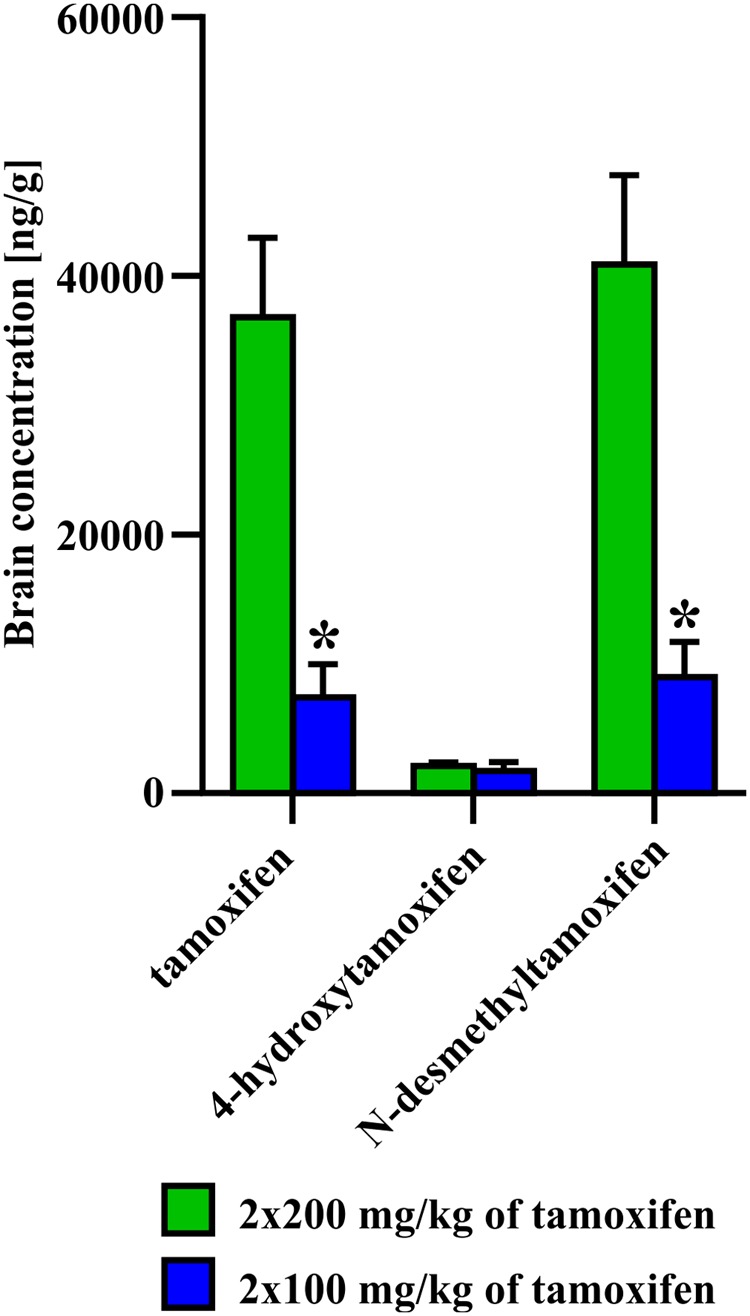
**Difference in concentration of 4-hydroxytamoxifen at day 0, after administration of 2 × 200 or 2 × 100 mg/kg of tamoxifen.** No difference in concentration of 4-hydroxytamoxifen, unlike those of tamoxifen and *N*-desmethyltamoxifen, suggests that the pathway toward the most potent metabolite is saturated already, when 2 × 100 mg/kg of tamoxifen i.p. was administrated. Values of *p* < 0.05 were considered significant (^∗^).

In the second set of experiments, we evaluated the influence of different doses of tamoxifen, as well as different dosing schemes on the degradation kinetics of tamoxifen and its metabolites in young adult C56BL/6J male mice. As a control, we used the application scheme of 2 × 200 mg/kg (high dose), the same as in the first experimental set, and then we tested 2 × 100 mg/kg, (low dose) 24 h apart and 5 × 100 mg/kg 24 h apart in 5 consecutive days. Eventually, we also tested the dose of 2 × 100 mg/kg in aged (over 15 months) C57BL/6J male mice. As expected, in the case of low doses of tamoxifen, the degradation kinetics were significantly faster when compared with that of high doses (**Figure 4**), while the recombination rate was preserved (Supplemental Figure S1C). Surprisingly, although the peak concentrations of tamoxifen and *N*-desmethyltamoxifen in the brain were significantly decreased when the low dose was used, the peak concentration of 4-hydroxytamoxifen in the brain remained unchanged, independent of initial tamoxifen dose (**Figure 5**). Regarding the aged mice, the lower dose of tamoxifen improved their survival rate to 100% and allowed them to degrade all substances within 8 days. The aged mice with the low dose of tamoxifen, showed the same degradation rate as young adult mice with the high dose of tamoxifen with exceptions for *N*-desmethyltamoxifen in time-point 2 days and endoxifen in time-point 4 days. Interestingly, the degradation kinetics of tamoxifen and its metabolites after single 200 mg/kg dose scheme was comparable with that of low dose; however, the single dose scheme resulted in lower recombination rate (Supplemental Figure S2)

**FIGURE 6:**
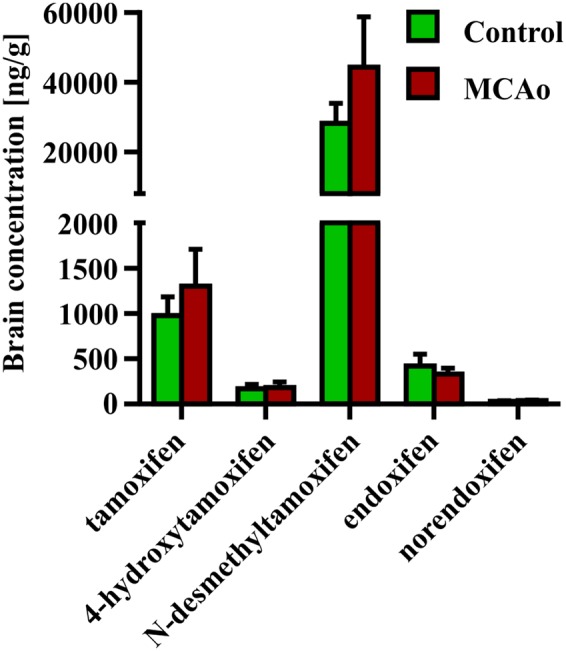
**Influence of blood-brain barrier disruption on brain concentration of tamoxifen and its metabolites.** Disruption of the blood-brain barrier by the middle cerebral artery occlusion, causes no changes in the ipsilateral hemisphere concentration of any tamoxifen metabolites.

Finally, we tested the impact of disruption of the blood-brain barrier caused by FCI on the concentration of tamoxifen and its metabolites. For this purpose, we performed FCI in young adult mice 3 days after the last tamoxifen treatment (2 × 200 mg/kg). The brain concentration of tamoxifen and its metabolites were analyzed 24 h after FCI induction, which corresponds to day 4 in control animals. Interestingly, no changes in the concentration of all substances were detected when compared to uninjured animals (**Figure 6**).

Taken together, tamoxifen metabolism in the brain depends on mouse strain, age of the animal and initial tamoxifen dose. Our results show that a low dose of tamoxifen results in sufficient 4-hydroxytamoxifen concentration in the brain, which promotes maximal recombination, and moreover, it is not lethal for aged mice with presumed tamoxifen metabolism limitations. Interestingly, although concentrations of tamoxifen and its metabolites in the brain varied significantly based on the age and dose and persisted in the brain for a long time, the blood concentrations were not dose- or age-dependent, with exception for tamoxifen 6 h after its administration (Supplemental Figure S3).

### Minimal Effective Concentration of 4-Hydroxytamoxifen in the Brain *In vivo*

Since the effective concentration of tamoxifen necessary to promote recombination *in vivo*, could hardly be determined due to its ongoing metabolism to form much more effective 4-hydroxytamoxifen, we determined the minimal effective brain concentration of 4-hydroxytamoxifen itself. 4-hydroxytamoxifen is metabolized to endoxifen, which is less effective than 4-hydroxytamoxifen, and therefore its influence on the 4-hydroxytamoxifen efficacy could be neglected.

**FIGURE 7:**
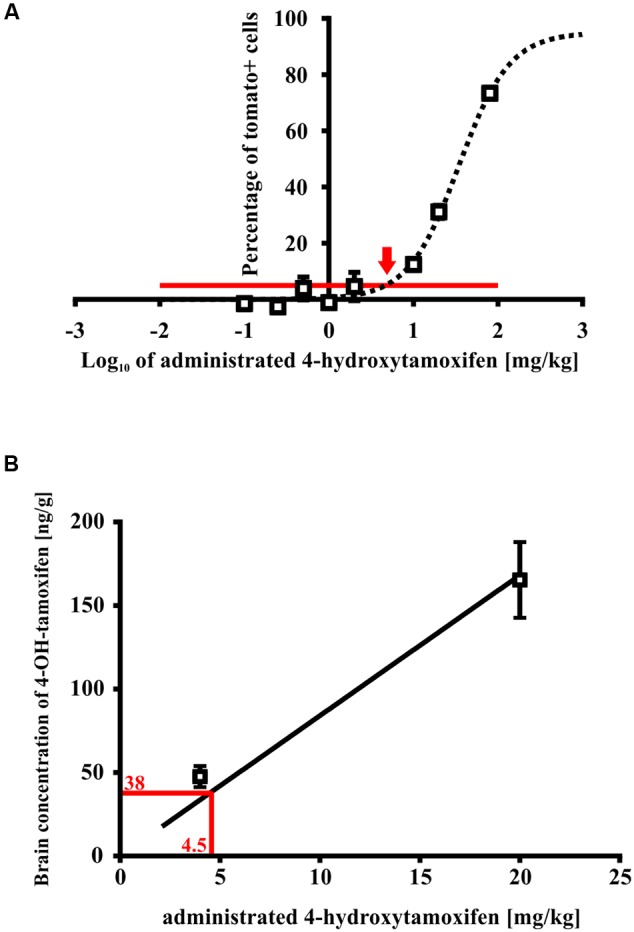
**Minimal effective 4-hydroxytamoxifen concentration in mouse brains. (A)** Spontaneous recombination takes place in 3% of cells (indicated by the red line), relative to the maximal recombination rate. An additional recombination over the spontaneous level was caused by the administration of 4-hydroxytamoxifen in concentration exceeding 4.5 ± 1.3 mg/kg (indicated by the red arrow), **(B)** which corresponds to 4-hydroxytamoxifen concentration of 38.0 ± 22.0 ng/g in the brain.

For this purpose, we treated Cspg4/tomato mice with a different concentration of 4-hydroxytamoxifen, and quantified the number of recombined tomato+ cells in fixed brain slices (Supplemental Figure S1D). From such analysis we obtained a relationship between the *number of tomato+ cells*, and *4-hydroxytamoxifen concentration*, which was fitted well by a sigmoidal curve, allowing us to calculate the spontaneous recombination rate, and minimal concentration of administrated 4-hydroxytamoxifen necessary to promote additional recombination above the spontaneous level (**Figure 7A**). The theoretical spontaneous recombination in Cspg4/tomato mice, calculated from the fitted curve, occurs in 5.0 ± 1.3% of cells from the maximal recombination rate observed, which correlates well with the spontaneous recombination rate obtained from non-treated animals (Supplemental Figure S1D). By subtracting the spontaneous recombination rate from the percentage of *tomato+ cells, it* enabled us to determine that the minimal effective concentration of administrated 4-hydroxytamoxifen capable of promoting recombination above the spontaneous level, is 4.5 ± 1.3 mg/kg (**Figure 7A**).

Next, we determined the peak concentration of 4-hydroxytamoxifen in the brain after administration of 5 and 20 mg/kg of 4-hydroxytamoxifen, using LC-MS in order to reveal the relationship between 4-hydroxytamoxifen concentration in the brain and concentration of administrated 4-hydroxytamoxifen. From the resulting values (47.5 ± 6.3 ng/g and 165.4 ± 22.7 ng/g for 5 and 20 mg/kg of administrated 4-hydroxytamoxifen, respectively) we obtained a linear relationship characterized with the equation *y* = 8.4x (**Figure 7B**). By substituting the *x*-value with the minimal effective concentration of administrated 4-hydroxytamoxifen, 4.5 ± 1.3 mg/kg, we calculated that the minimal effective concentration of 4-hydroxytamoxifen in the brain, which is necessary to promote recombination *in vivo*, was 38.0 ± 22.0 ng/g (**Figure 7B**).

In conclusion, the concentration of 4-hydroxytamoxifen in the brain decreases to the ineffective level within 6 days in the case of high doses, and within 4 days in the case of low doses in young adult mice (**Figure 4**).

## Discussion

To prevent undesirable recombination caused by residual concentrations of tamoxifen and its metabolites in the mouse brain, it is important to know the time window that allows their complete degradation. Moreover, it is also essential to obtain the highest possible recombination rate with minimal animal mortality in Cre-loxP systems, which should lead to cell-type specific gene knockout. Therefore, in this study we identified the minimal effective concentration of the most potent tamoxifen metabolite4-hydroxytamoxifen, which can promote recombination in the brains of C56BL/J mice. After determining the degradation kinetics of tamoxifen and its metabolites, we disclosed that all metabolites are degraded within 8 days when the dose of 2 × 200 mg/kg of tamoxifen is used. Furthermore, we revealed that the degradation time depends strongly on the initial dose of tamoxifen, since 6 days are sufficient for complete degradation of 2 × 100 mg/kg tamoxifen administration. Mouse age also plays an important role, because aged mice showed a high mortality rate after 2 × 200 mg/kg of tamoxifen administration, and they were unable to degrade tamoxifen or its metabolites within 6 days. Therefore, we conclude that a low dose of tamoxifen is sufficient to promote full recombination, and has minimal toxicity even in aged mice. In addition, we showed that disruption of the blood-brain barrier by FCI has no impact on the brain concentrations of tamoxifen and its metabolites.

### Effectivity of Tamoxifen and Its Metabolites to Promote Recombination

So far, the potency of tamoxifen and its metabolites was assessed in relation to their anti-estrogenic effects, primarily in tumor cell lines (Katzenellenbogen et al., 1984; Desta et al., 2004; Lim et al., 2005; Jordan, 2007). Here we tested the affinity of tamoxifen and its metabolites to cre-ER^TM^ (cre-Esr1^∗^) (Hayashi and McMahon, 2002), which corresponds to their ability to trigger recombination in our Cre-loxP system. Similarly to their anti-estrogenic effects (Katzenellenbogen et al., 1984; Desta et al., 2004; Lim et al., 2005; Jordan, 2007), the highest potency to bind cre-ER^TM^ and thus to promote recombination, exhibited 4-hydroxytamoxifen. Tamoxifen and endoxifen showed 55-times and 4-times lower potency to bind cre-ER^TM^ than 4-hydroxytamoxifen, respectively (**Figure 1**). Although it was shown that *N*-desmethyltamoxifen displays similar biological activity as tamoxifen regarding their anti-estrogenic effects (Desta et al., 2004; Lim et al., 2005; Jordan, 2007), we found that *N*-desmethyltamoxifen is ineffective in its ability to bind and to activate cre-ER^TM^. This discrepancy is probably caused by structural differences between the estrogen receptor and ER^TM^, which is a mutant form of the ligand-binding domain of the estrogen receptor (Hayashi and McMahon, 2002). Norendoxifen, so far studied mostly for its aromatase-inhibiting properties (Lu et al., 2012), showed twice higher potency compared to tamoxifen.

### Degradation Period of Tamoxifen and Its Metabolites in the Mouse Brain

We assessed the i.p. administration of tamoxifen since the other possibilities of tamoxifen administration are not so reliable. Administration via drinking water is limited by the solubility of tamoxifen in the water and a long period of administration necessary to achieve the satisfactory recombination. The oral gavage seems to be more stressful for animals and moreover it is more time-consuming than i.p. administration. The i.p. administration is highly reproducible and easy to perform. According to Lien et al. (1991), the peak concentration of tamoxifen and its metabolites in the brain was measured 6 h after the last tamoxifen injection. Tamoxifen and *N*-desmethyltamoxifen showed the highest brain accumulation after two intraperitoneal tamoxifen applications. 4-hydroxytamoxifen showed 20-fold lower concentration in the brain, while endoxifen and norendoxifen did not even reach 1% of tamoxifen and *N*-desmethyltamoxifen concentration levels. This observation is partly in contrast with the work of Lien et al. (1991) and Iusuf et al. (2011), who found that *N*-desmethyltamoxifen and 4-hydroxytamoxifen brain concentration is twice lower compared to tamoxifen concentration in the brain. Such discrepancies were probably caused by different types of tamoxifen administration. While we used two intraperitoneal doses, Lien et al. (1991) and (Iusuf et al. (2011) administrated tamoxifen orally in one, three or fourteen doses, thus incorporating the gastro-intestinal tract into tamoxifen biotransformation. Moreover, Lien et al. (1991) used rats instead of mice in their work. On the other hand, consistently with Lien et al. (1991) and (Iusuf et al., 2011) we detected only traces of endoxifen, which is caused by its poor ability to cross the blood-brain barrier (Iusuf et al., 2011).

The complete degradation of tamoxifen and its metabolites, after the dose of 2 × 200 mg/kg, occurred within 8 days in young adult C57BL/6J mice with an exception for *N*-desmethyltamoxifen, which did not reach undetectable brain concentration within 8 days. Although we did not detect any differences in the degradation of tamoxifen and its metabolites between males and females, we did so between different mouse strains (**Figure 3**). FVB/NJ mice had significantly lower concentration of tamoxifen and its metabolites in the brain in several time-points when compared to C57BL/6J mice. These results point to strain-dependent differences in the metabolic processes, which were documented previously by Berglund et al. (2008) and Vaillant et al. (2014). Besides the strain-dependent differences, we also observed a major age-related alteration in the degradation of tamoxifen and its metabolites. In agreement with observations that metabolic rate decreases with aging (Houtkooper et al., 2011), aged mice were unable to degrade the tamoxifen and its metabolites within 6 days (**Figure 3**). This often led to the death of aged animals prior to the final time-point of our experiments.

We also tested the ability of mice to degrade tamoxifen and its metabolites after the administration of a lower dose (2 × 100 mg/kg of tamoxifen). Obviously, lower tamoxifen doses led to significantly faster degradation of all tamoxifen metabolites within 6 days (**Figure 4**). Interestingly, the peak concentration of 4-hydroxytamoxifen in the brain reached the same level independently of the initial tamoxifen dose (**Figure 4**). Given that tamoxifen and *N*-desmethyltamoxifen peak concentrations in the brain were significantly lower when a low dose of tamoxifen was used, we hypothesize that the mechanisms responsible for transformation of tamoxifen into 4-hydroxytamoxifen in the brain are fully saturated already, when the dose of 2 × 100 mg/kg is used. When such a phenomenon is taken into account, using tamoxifen in the total dose higher than 200 mg/kg is unnecessary, since it might only lead to higher metabolic stress and does not increase the recombination rate. A lower dose of tamoxifen improved the survival rate of aged mice; however, their metabolic deficits were still evident.

Permanent MCAo resulting in FCI also leads to disruption of the blood brain barrier, and over-perfusion of the infarcted site (Yan et al., 2015). Hence, we expected that FCI should affect concentration of tamoxifen and its metabolites in the brain. Since we detected no changes in the brain concentration of any tamoxifen metabolites after FCI, we conclude that this type of injury has no impact on tamoxifen metabolism. The subsequent analyzes showed that the blood concentrations of tamoxifen and its metabolites at day 4 were very low and therefore the disrupted blood brain barrier and over-perfusion did not change the concentrations in the brain tissue.

### Implications of Tamoxifen Metabolism in the Brain

The knowledge of degradation kinetics of tamoxifen and its active metabolites in the mouse brain is of big importance, especially concerning genetic fate-mapping using the tamoxifen inducible cre-loxP system. It is important to use the precise amount of administrated tamoxifen, which causes a sufficient recombination rate, and simultaneously, to avoid excessive metabolic stress of animals. Furthermore, the proper time window for allowing the complete degradation of active tamoxifen metabolites has to be applied in order to prevent possible data misinterpretation (labeling of desired cells is triggered by the expression of cell-type specific marker under physiological condition. Many of these cell-type specific markers become widely expressed also in other cell types after pathologic stages, treatments, aging etc. and therefore the presence of tamoxifen and its metabolites residues can cause recombination in non-traced cell types). In addition, the animals age has to be taken into account during experiment design, since the aged animals posses some limits in tamoxifen and its metabolites degradation, and therefore require specific treatment. Moreover, a wide use of tamoxifen in human medicine could bring other implications, in which the age of patients and application scheme should be taken into account.

## Author Contributions

MA, MV, and PH designed and conceptualized all the experiments. MV, PH and DK performed and analyzed all the *in vitro* experiments, prepared all tissue samples and performed MCAo operations in this project. ZK performed and analyzed LC-MS experiments. MV and PH performed the overall analysis of results from these experiments. MV, MA and PH wrote and edited the manuscript.

## Conflict of Interest Statement

The authors declare that the research was conducted in the absence of any commercial or financial relationships that could be construed as a potential conflict of interest.
