# Supplementary figures and images for: Tamoxifen in the Mouse Brain: Implications for Fate-Mapping Studies Using the Tamoxifen-Inducible Cre-loxP System
Source: Front Cell Neurosci. 2016 Oct 20;10:243. doi: 10.3389/fncel.2016.00243 (PMC5071318; doi:10.3389/fncel.2016.00243)

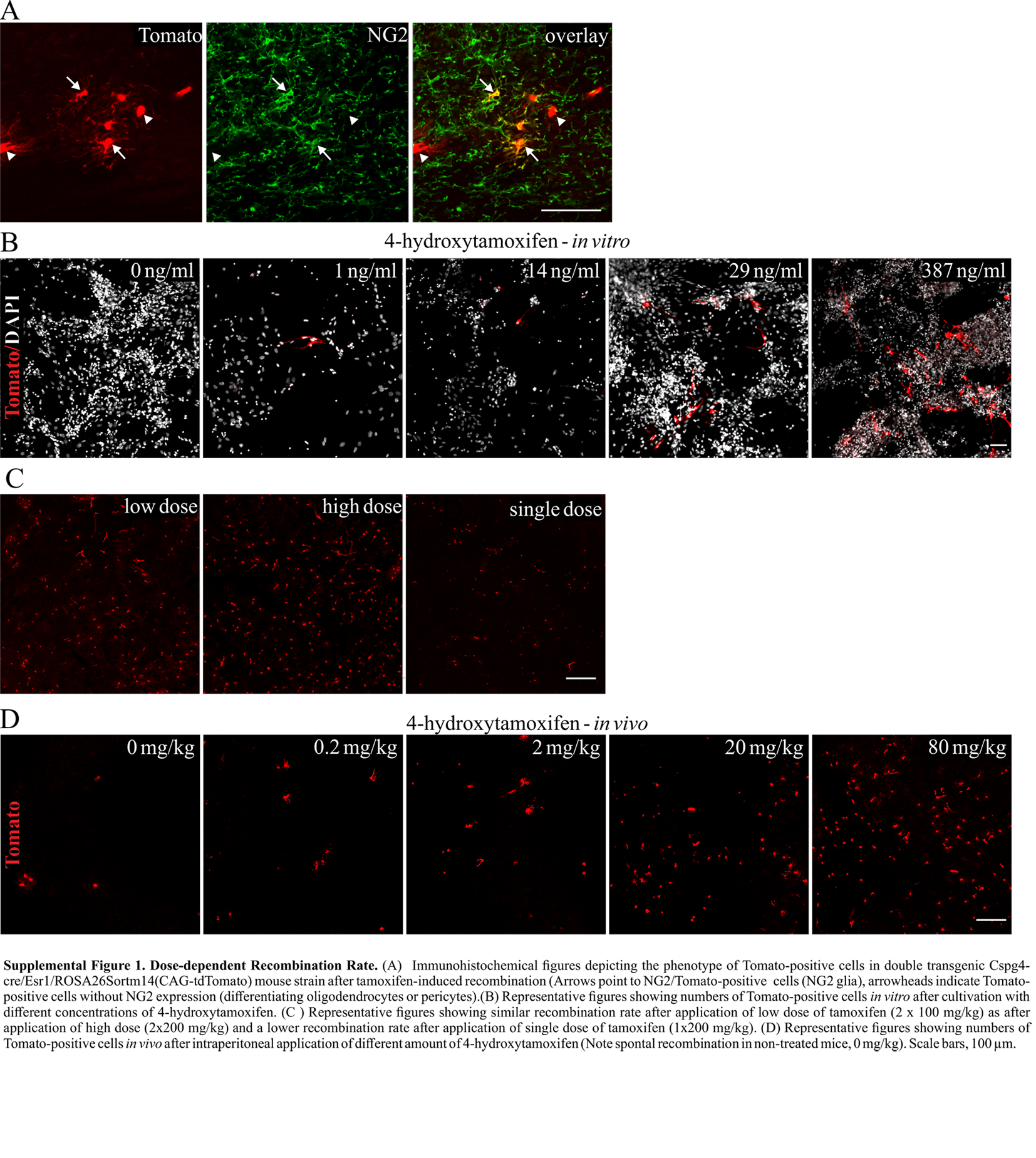

Supplement: Supplementary file 1 [file Image_1.TIF]

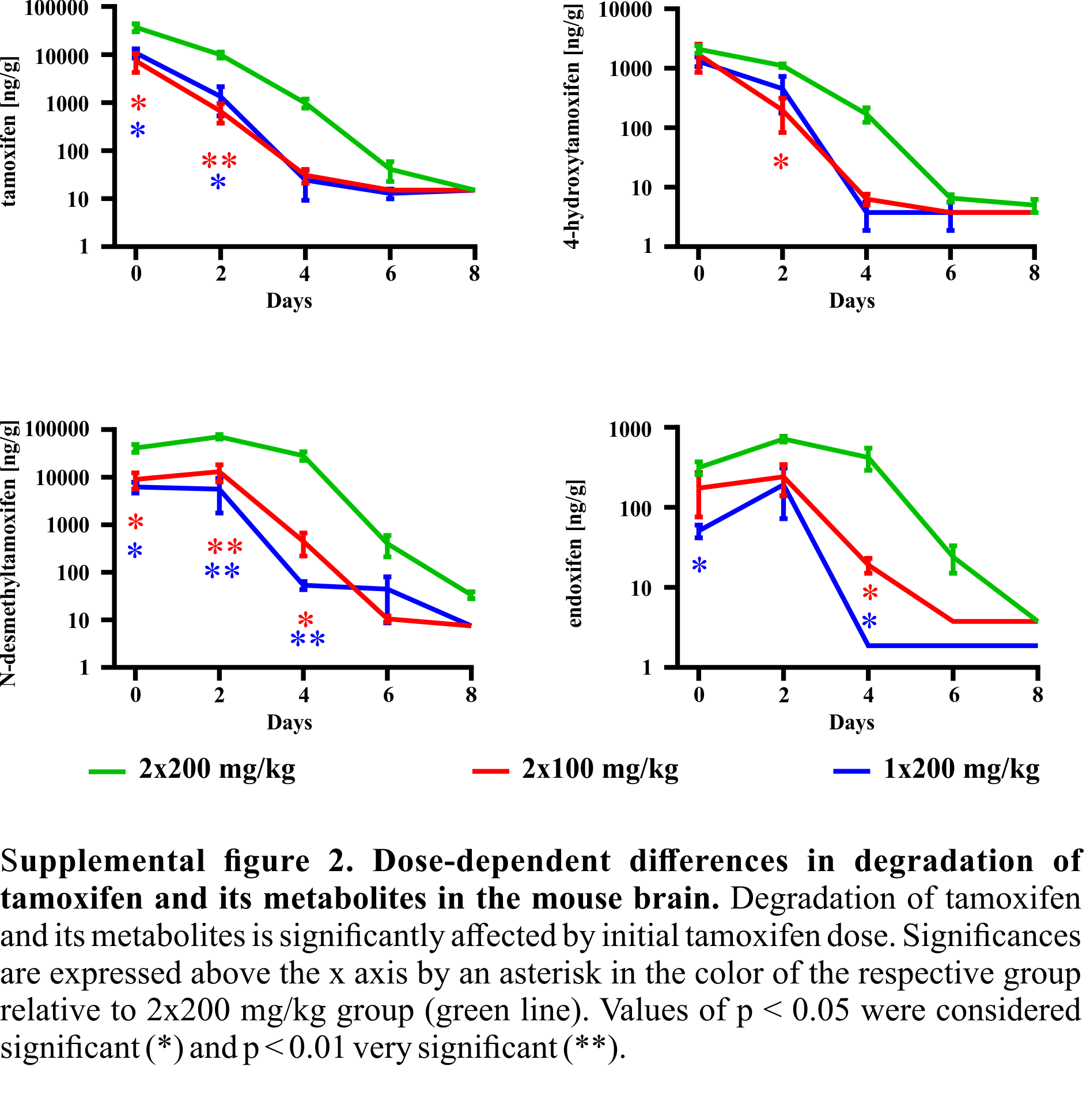

Supplement: Supplementary file 2 [file Image_2.TIF]

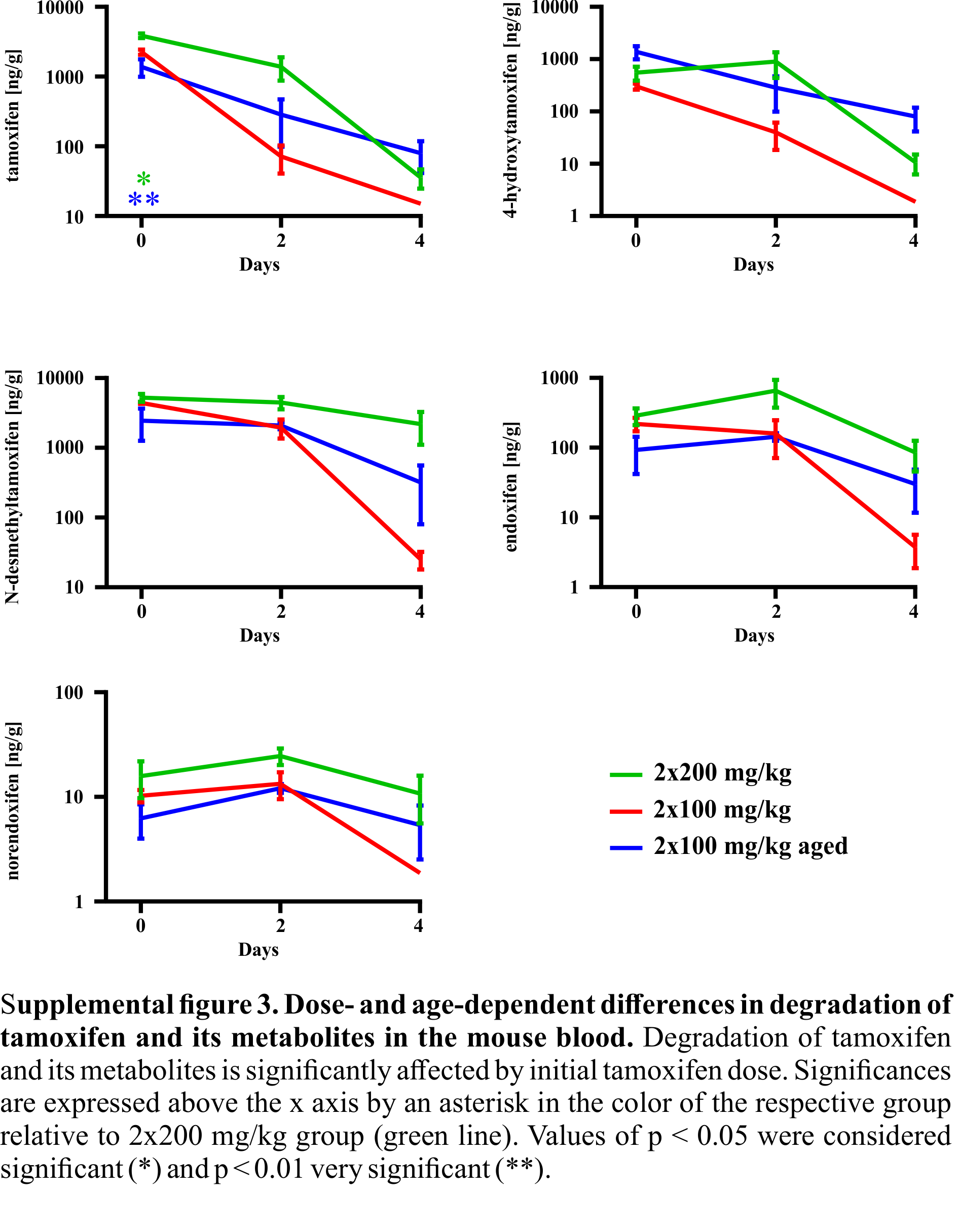

Supplement: Supplementary file 3 [file Image_3.TIF]
